# IκB kinases increase Myc protein stability and enhance progression of breast cancer cells

**DOI:** 10.1186/1476-4598-10-53

**Published:** 2011-05-16

**Authors:** Pei-Yen Yeh, Yen-Shen Lu, Da-Liang Ou, Ann-Lii Cheng

**Affiliations:** 1 Department of Oncology, National Taiwan University Hospital, No. 7, Chung-Shan South Road, Taipei, 100, Taiwan; 2 National Center of Excellence for Clinical Trial and Research, College of Medicine, National Taiwan University, No1, Jen Al Road Section1, Taipei, 100, Taiwan; 3 Department of Internal Medicine, National Taiwan University Hospital, No. 7, Chung-Shan South Road, Taipei, Taipei, 100, Taiwan; 4 Graduate Institute of Oncology, College of Medicine, National Taiwan University, No1, Jen Al Road Section1, Taipei, 100, Taiwan

## Abstract

**Background:**

Both IκB kinase (IKK) complex and oncgenic protein Myc play important roles in cancer progression, including cancer cell invasiveness and metastasis. The levels of Myc is regulated by the phosphorylation of Myc at Thr58 and Ser62.

**Results:**

In this study, we show that the expression of Myc is associated with IKKα and IKKβ in breast cancers and that Myc is an IKKs substrate. Suppression of IKK activity by either chemical inhibitor or transfection of kinase-dead mutants decreases the phosphorylation of Myc at Ser62 and enhances the degradation of Myc. Consequently, these treatments decrease the tumorigenic and invasive ability of breast cancer cells. Furthermore, doxorubicin, a frequently used anticancer drug in breast cancer, activates IKKs and Myc, thereby increasing invasiveness and tumorigenesis of breast carcinoma MCF7 cells. Inhibition of IKKs prevents these doxorubicin-induced effects.

**Conclusions:**

Our study indicates that IKKs tightly regulate Myc expression through prolonging protein stability, and suggests that IKKs are potentially therapeutic targets and that suppression of IKKs may be used following chemotherapy to reduce the risk of treatment-induced tumor progression.

## Background

The IKK complex is composed of two kinase catalytic subunits IKKα and IKKβ and a non-kinase scaffold protein IKKγ [1-3]. The complex functions as an upstream kinase involved in the activation of nuclear factor kappa B(NF-κB)by phosphorylation of the NF-κB inhibitory molecule, IκBα, resulting in the subsequent degradation of IκBα through the ubiqutin/proteasome pathway. The released NF-κB translocates into the nucleus and then regulates the expression of multiple genes [1,4,5]. Numerous reports have indicated that the functions of IKKs are necessary for cancer cell survival and progression [3,6-8].

Most studies regarding IKKs are actually focused on their downstream molecule, NF-κB, and the thinking that IKKs might be therapeutic targets is trying to indirectly suppress NF-κB activation [1,9]. However, accumulating evidence has indicated that IKKs have NF-κB- independent effects on multiple proteins [1,10]. For example, IKKβ phosphorylates tumor suppressor FOXO3a, and consequently induces FOXO3a nuclear exclusion and degradation, thereby promoting tumor survival [11]. Interesting, IKKα and IKKβ may have opposite effect on certain proteins. For example, IKKα increases but IKKβ decreases the transcriptional activity and protein level of β-catenin [12,13]. The biological significance of IKKs is getting complicated and requires further characterization. The identification of new substrates of IKKs is important for the understanding of IKKs functions in cancer biology.

The oncogenic Myc protein is a transcription factor that regulates a wide spectrum of downstream genes involved in cancer cell metabolism, growth, and progression [14-17], and it is well documented that Myc plays an important role in breast cancer metastasis [17-19]. Abnormal expression of Myc is frequently associated with cancer progression [20-23]. Several transcription factors, including NF-κB, E_2_F, STAT, and β-catenin, are involved in the regulation of Myc expression [24,25]. Inhibition of these transcription factors suppresses cancer cell survival in part by decreasing Myc expression.

The Myc protein level is further regulated by control of protein stability, which is determined by a complicated protein kinase/phosphatase system. Phosphorylation of Myc at Ser62 increases protein stability. The kinases ERK (extracellular signal-regulated kinase), JNK (c-Jun N-terminal kinase) and cdk1 (cyclin-dependent kinase 1) have been identified to phosphorylate Myc at Ser62 [16,26,27]. The Ser62 phosphorylated Myc is further phosphorylated at Thr58 by glycogen synthase kinase 3β. The Thr58/Ser62 dual phosphorylated Myc is acted on by protein phosphatase 2A [PP2A] to dephosphorylate Ser62. Then, monophosphorylated Myc (at Thr58) is degraded by ubiquitin/proteosome system. A cellular PP2A inhibitor cip2A which is overexpressed in several cancers has been shown to increase Myc levels via suppression of PP2A activity [16,28,29]. Given the fact that numerous intra- and extra-cellular stimuli regulate the activation of Myc, it is expected that other unidentified kinases may be also involved.

In this study, we investigated the association of Myc and IKK/NF-κB in breast cancer. Interestingly, IHC staining of breast cancer specimens showed that the expression of Myc was closely associated with that of IKKs but not with NF-κB p65. We demonstrated that IKKα and IKKβ increased Myc protein levels by prolonging protein stability, and this consequently promoted the tumorigenic and invasive activity of breast cancer cells. Our results also indicated that IKKα but not IKKβ directly interacted with Myc. In addition, we showed that a conventional anti-cancer drug, doxorubicin, activated the IKKs-Myc pathway which might enhance tumor progression. Together, our study indicated that suppression of IKKα and IKKβ may decrease basal and stress-induced Myc protein levels. The latter suggested that inhibition of IKKs may be used to block treatment-induced tumor progression.

## Materials and methods

### Patients

The specimens were acquired between 2009 and 2010 from patients with infiltrating ductal carcinoma of the breast, prior to chemotherapy without adjuvant, and were kindly provided by the Department of Pathology, National Taiwan University Hospital, on the basis of their availability. Use of these tissue materials followed the regulations of the research ethics committee of the National Taiwan University Hospital.

### Immunohistochemical study (IHC)

The tumor tissue embedded in paraffin was cut in 5-μM section, and then de-paraffinized in xylene and rehydrated. For antigen-retrieval, the sections were incubated with 10 mM sodium citrate buffer (pH 6.0) in a boiling water bath for 15 minutes. The slides were then incubated with 3% H_2_O_2 _in methanol to block endogenous peroxidase activity. IHC staining was performed using a streptavidin-biotin-peroxidase kit(Vectastain Universal Quick Kit; Vector Laboratories, Burlingame, CA, USA) according to the manufacturer's instructions. DAB/chromogen system (Dako Northern America, Inc.)was used to develop the image. The antibodies, including anti-IKKα (sc-7183), anti-IKKβ (sc-7329), anti-NF-κB p65 (sc-372) and anti-Myc (sc-40), were purchased from Santa Cruz Biotechnology (Santa Cruz, CA, USA) and were used at a 1:50 dilution. The staining was judged and counted by a researcher who was blinded regarding the corresponding patient.

### Cells and reagents

Breast cancer cell line MCF7 was purchased from the American Type Culture Collection. The cells were cultured in Dulbecco's Modified Eagle Medium supplemented with 10% FCS and incubated in 37°C with 5% CO_2_. All chemicals and reagents used were purchased from Calbiochem or Sigma-Aldrich.

### Plasmid and transfection

The IKKα and IKKβ (both wild-type and kinase-dead mutant) expression vectors were kindly provided by Professor WC Greene (Gladstone Institute of Virology and Immunology, University of California, San Francisco), and were used to transfect MCF7 cells using Lipofectamine 2000 (Invitrogen, Carlsbad, CA, USA). The transfected cells were selected and maintained with complete medium containing 500 μg/ml G418.

### Coimmunoprecipitation and Western blot analysis

Whole cell lysates were prepared in RIPA solution containing a cocktail of protease and phosphatase inhibitors, or the cells were fractionated into cytoplasmic and nuclear fractions. For cellular fractionation, the cells were harvested and resuspended in hypotonic solution (Buffer 1; 1 mM KCl, 0.2 mM MgCl_2_, 4 mM Tris, pH7.6, containing a cocktail of protease inhibitors) for 20 minutes on ice. The cells were then lysed by adding lysis buffer (Buffer 1 containing 1% Triton X-100) and vigorously vortexed for 20 seconds, and then centrifuged at 1500 rpm for 5 minutes. The supernatant was collected as cytoplasmic fraction, and the pellet was washed once with PBS buffer and then lysed by RIPA buffer as nuclear fraction. After determination of protein concentration of each lysates, the aliquots (15 μg) were subjected to Western blot analysis. For coimmunoprecipitation, target proteins were immunoprecipitated from whole cell lysates (500 μg) by adding 2 μg antibody at 4°C overnight followed by protein A/G agarose adsorption(Santa Cruz Biotechnology, Santa Cruz, CA, USA). The complex was washed once with RIPA buffer containing 500 mM NaCl, once with RIPA buffer containing 250 mM NaCl, and two times with RIPA buffer. The washed complex was then resolved in SDS-sample buffer and was subjected to Western blot analysis. The antibodies used were as follows: anti-NF-κB p65, anti- NF-κB p50, anti-pMyc, anti-Max, and anti-twist antibodies from Santa Cruz Biotechnology; anti-IKKα, anti-IKKβ, anti-Myc, and anti-cyclin D1 antibodies from Cell Signaling; anti-pS62 Myc antibody from Abnova (Taipei City, Taiwan); and anti-pT58 Myc antibody from Abgent(San Diego, CA, USA).

### Confocal microscopy observation

Breast cancer tissue slides previously identified as positive or negative staining by IHC analysis were used for confocal microscopy observation. The process for de-paraffin and antigen retrieval was as in the IHC staining protocol. The slides were dual-stained using rabbit anti-IKKα and mouse anti-Myc antibodies coupled with FITC-conjugated goat anti-rabbit IgG and rodamine-conjugated donkey anti-mouse IgG antibodies, or goat anti-IKKβ and mouse anti-Myc antibodies coupled with FITC-conjugated donkey anti-goat IgG and rodamine-conjugated donkey anti-mouse IgG antibodies. The images were captured using a confocal microscope (TCS SP2; Leica, Wetzlar, Germany) at the confocal microscopy core-facilities of the National Taiwan University Hospital.

### Quantitative RT-PCR

RNA was extracted using Trizol reagent (Invitrogen). cDNAs were synthesized by a reverse transcription reaction. The expression of gene was quantified using SYBR Green PCR Master Mix on an ABI PRISM 7900 system(Applied Biosystems). The primers used were 5'-TCGACTACGACTCGGTGCAG (forward), 5'-TGGGCAGCAGCTCGAATTTC (reverse) for Myc; and 5'-TCGGAGTCAACGGATTTGG(forward), 5'-GAATTTGCCATGGGTGGAAT (reverse) for GAPDH. The expression level of GAPDH was used a control. The PCR reaction was performed with the following program: 95°C for 10 minutes, and then 40 cycles of 95°C for 15 seconds and 60°C for 1 minute. The relative expression level of the target gene was calculated using the ΔCt (threshold cycle) method: relative expression = 2^-ΔCt^, where ΔCt = Ct (target gene) - Ct (control gene).

### Determination of RNA and protein stability

To determine the stability of mRNA, the cells were treated with 5 μg/ml actinomycin D to block new mRNA synthesis. Total RNAs were extracted at different time point after treatment. Random-primed reverse transcribed cDNA was subjected to qPCR analysis to determine the relative expression levels of the Myc and GAPDH genes. For protein stability analysis, the cells were treated with 10 μM cycloheximide, and then whole cell lysates were prepared after different durations. The lysates (15 μg) were then subjected to Western blot analysis to identify the Myc and tubulin proteins. Protein levels were quantified using VisionWorksLS version 7.0 software (UVP, Upland, CA, USA).

### Assay of growth rate and colony formation in soft agar

For proliferation assay, the cells (2000 cells/well) were seeded into 96-well culture plate. The cell number was evaluated by a 3-(4,5-dimethylthiazol-2-yl)-2,5-diphenyltetrazolium bromide (MTT)-based semi-automated colorimetric assay. For soft agar colony-forming assay, 20000 cells in complete medium containing 0.3% Bacto-agar were overlaid on 1% agar-complete medium in 60 mm culture dish for two weeks. The colony number was counted under phase contrast microscopy at 4X magnification.

### Invasion assay

An *in vitro *invasion assay was performed by analyzing the ability of tumor cells to penetrate through Matrigel (BD Biosciences). The cells (2 × 10^4) ^were seeded into the chamber of a 24 trans-well plate preloaded with 0.1 ml Matrigel for 16 hours. The penetrated cells were fixed with methanol, stained with Giemsa solution, photographed, and counted.

### Statistical analysis

The association of IKKα, IKKβ, NF-κB p65 and Myc expression in a total of 21 breast cancer specimens was analyzed with Fisher exact test (SPSS software for Windows 11.0, SPSS, Inc., Chicago, IL). A probability of error <5% was regarded as significant.

## Results

### The expression of Myc is associated with IKKs but not with NF-κB in breast cancer

**Figure 1:**
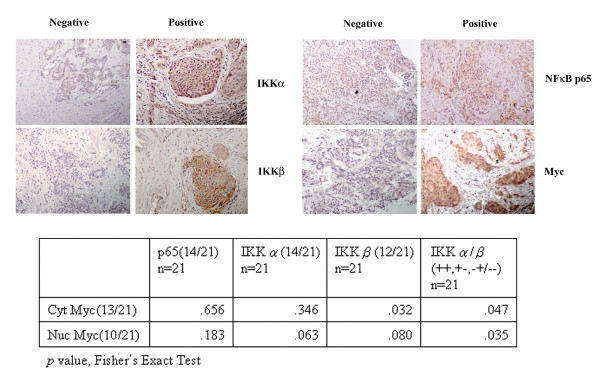
**Myc expression level is associated with IKKα and IKKβ but not NF-κB**. IHC staining was used to identify the expression of IKKα, IKKβ, NF-κB p65 and Myc in breast cancer specimens (upper panel). Representative pictures of positive and negative staining are shown. Statistic analysis shows a positive association among IKKα and/or IKKβ and Myc expression in 21 breast cancer specimens (lower panel).

To identify whether the expression of Myc was associated with IKKs/NF-κB expressions *in vivo*, IHC staining was used to identify the expression of IKKα, IKKβ, Myc and NF-κB p65 in a total of 21 breast cancer specimens. The staining of more than 50% of the cells in a single field of view and at least 5 fields in a specimen were designated as positive [Figure 1]. The ratio of positive staining was 67% (14/21) for IKKα, 57% (12/21) for IKKβ, 67% (14/21) for NF-κB p65, and 62% (13/21) for Myc. When the expression of Myc was assessed based on nuclear staining, the ratio was 48% (10/21), similar to the result of a previous study [30]. Statistical analysis showed that Myc expression was correlated with IKKα and IKKβ expression, whereas it had no correlation with the expression of NF-κB p65 (Figure 1). This result suggested that IKKs might regulate Myc expression through an NF-κB-independent pathway.

### Suppression of IKK activity decreases Myc protein levels in MCF7 cells

**Figure 2:**
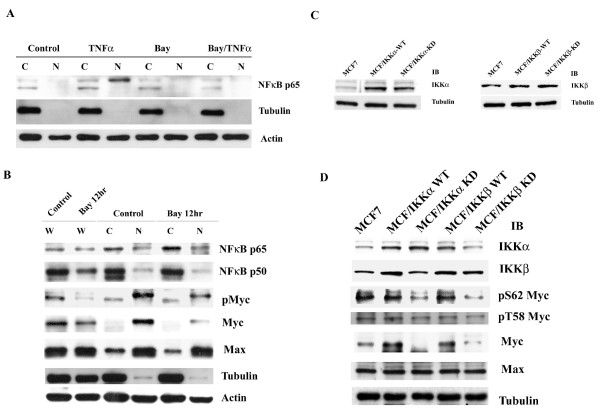
**IKKα and IKKβ increase Myc protein level**. (A). MCF7 cells were treated with 10 μM Bay11-0782 for 12 hours and then were further treated with 20 ng/ml TNF-α for 30 minutes. The cytoplasmic and nuclear extracts were subjected to Western blot analysis of NF-κB p65 subcellular distribution. Stains of tubulin were used to represent the clearance of cellular fractionation and stains of actin were used as loading control. (B). MCF7 cells were treated with 10 μM Bay11-0782 for 12 hours, and then the whole cell lysates, the cytoplasmic and nuclear extracts were prepared. Equal amount of proteins (15 μg/lane) were subjected to Western blotting analysis. (C). MCF7 cells were transfected with IKKα and IKKβ (both wild-type and kinase-dead mutant). The transfected cells were analyzed by Western blotting of IKKα and IKKβ.(D). The whole cell lysates prepared from indicated cells were subjected to Western blot analysis.

To explore the effects of the IKKs on Myc expression, an IKK inhibitor Bay11-7082 [31,32] was used to treat breast carcinoma MCF7 cells. Bay11-0782 blocked TNFα-induced NF-κB p65 nuclear translocation (Figure 2A), whereas it did not alter the basal level of cytoplasmic or nuclear NF-κB p65 or p50, suggesting that Bay11-7082 did not influence basal activity of NF-κB. Bay11-7082 markedly decreased the level of phosphorylated and total Myc protein. The Myc-binding partner, Max, was not affected by Bay11-7082 (Figure 2B).

To distinguish the role of IKKα and IKKβ in the regulation of Myc expression, MCF7 cells were transfected with IKKα or IKKβ [either wild-type or kinase-dead mutant] expression vectors (Figure 2C). Wild-type IKKα or IKKβ increased Myc protein levels, on the other hand, kinase-dead IKKα or IKKβ decreased Myc expression. Further, Western blot analysis showed that while the phosphorylation of Myc at Ser62 was increased in wild-type IKKα- and IKKβ-transfected cells and decreased in kinase-dead mutant transfected cells, the phosphorylation of Myc at Thr58 was not affected in any of the transfected or control cells. The expression of Max was not changed by the manipulation of IKKs (Figure 2D). Furthermore, we observed a reciprocal upregulation of IKKα and IKKβ (Figure 2D).

### Suppression of either IKKα or IKKβ slows the degradation rate of Myc mRNA

**Figure 3:**
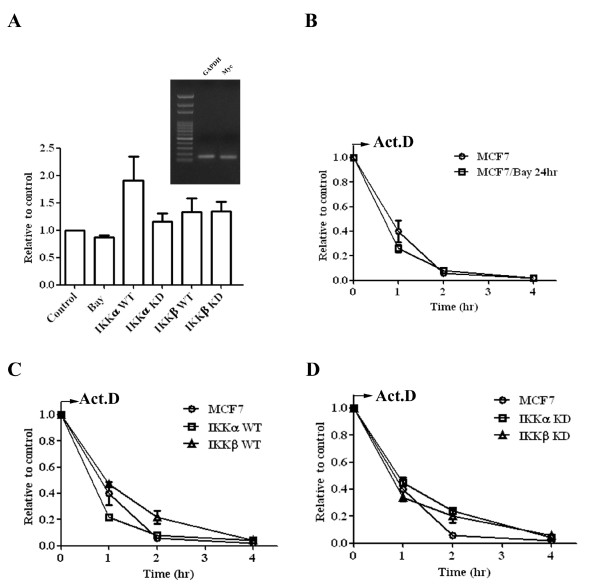
**IKKα and IKKβ increase Myc in a transcription-independent manner**. **(**A) MCF7 cells were treated with 10 μM Bay11-0782 for 12 hours. The RNA was extracted from indicated cells and random-primed reverse transcribed into cDNA. The relative expression of Myc was determined by qPCR. The expression of GAPDH was used as an internal control. The specificity of the primer set for Myc and GAPDH was demonstrated by agarose electrophoresis of PCR product (inserted figure) and by analysis of dissociation curve (data not shown). Each data represents mean ± SD calculated from two independent experiments. To determine the degradation rate of Myc mRNA, the cDNAs were prepared from (B) MCF7 cells were treated with 10 μM Bay11-0782 for 12 hours, (C) MCF7, wild-type IKKα and IKKβ transfected cells, and (D) MCF7, kinase-dead IKKα and IKKβ transfected cells. The relative level of Myc mRNA was determined by qPCR and expressed along a time course after adding actinimycin D. Each data represents mean ± SD calculated from two independent experiments.

To identify whether IKKα or IKKβ could increase the transcription of Myc mRNA, qPCR was used to determine the relative levels of Myc mRNA in Bay11-7082 treated MCF7 cells and IKK-transfected cells (Figure 3A). Overexpression of either wild-type IKKα or IKKβ slightly increased the level of Myc mRNA. The induction level was less than two-fold of the control which is the cut-point of most gene array analyses. Interestingly, while Bay11-7082 only marginally reduced Myc mRNA levels, a small increase of Myc mRNA (less than 1.5-fold of control levels) was observed in both kinase-dead IKKα- and IKKβ-transfected cells. These results suggested that IKKs could increase Myc protein levels in a transcription-independent manner.

To explore the mechanism which directs the increase of Myc mRNA in kinase-dead IKKα- and IKKβ -transfected cells, we hypothesized that the degradation rate of Myc mRNA might be prolonged in response to the decreased Myc protein levels. We used actinomycin D to block new mRNA synthesis and then determined the degradation rate of the Myc mRNA by qPCR. The decay of Myc mRNA was at a comparable rate among Bay11-7082 treated MCF7 cells and wild-type IKKα- and IKKβ-transfected cells, whereas a prolonged degradation rate was observed in kinase-dead IKKα- and IKKβ-transfected cells (Figure 3B, C and 3D).

### IKKs increase Myc protein stability

**Figure 4:**
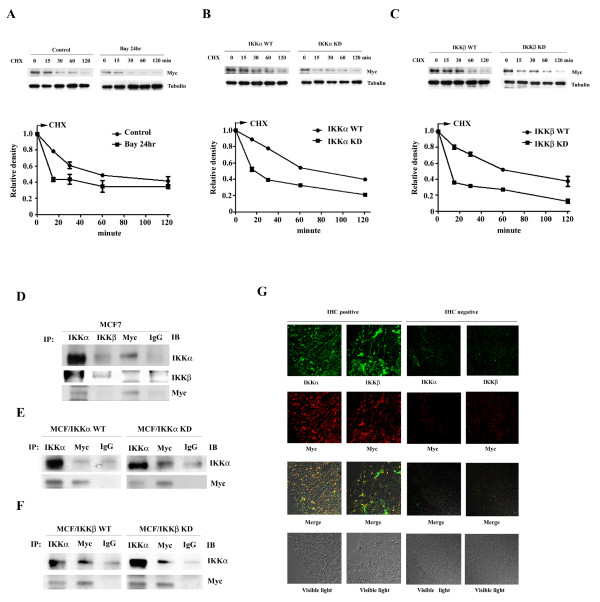
**IKKα and IKKβ increase Myc protein stability**. The stability of Myc protein was analyzed by Western blot analysis of the whole cell lysates prepared from (A) MCF7 with or without a 12 hours, 10 μM Bay11-0782 treatment (B) wild-type and kinase-dead IKKα transfected cells (C) wild-type and kinase-dead IKKβ transfected cells along a time course after adding cycloheximide. The stain of tubulin was used as loading control. The reading of Myc density was normalized to the reading of tubulin density. Each data represents mean ± SD calculated from two independent experiments. Whole cell lysates were prepared from (D) MCF7 cells, (E) wild-type and kinase-dead IKKα transfected cells, and (F) wild-type and kinase-dead IKKβ transfected cells were subjected to coimmuoprecipitation using indicated antibodies. The precipitated complex was further Western blot analyzed the corresponding proteins shown in the figure. (G) Confocal microscopy observation. Breast cancer tissues which were previously identified positive or negative for IKKα, IKKβ and Myc expressions by IHC staining were used. The slides were dual stained with rabbit anti-IKKα/mouse anti-Myc antibodies coupled with FITC-conjugated goat anti-rabbit IgG/Rodamine-conjugated donkey anti-mouse IgG antibodies or goat anti-IKKβ/mouse anti-Myc antibodies coupled with FITC-conjugated donkey anti-goat IgG/Rodamine-conjugated donkey anti-mouse IgG antibodies, respectively.

Next, we determined the degradation rate of the Myc protein in Bay11-0782 treated MCF7 cells and in IKK- transfected cells by Western blot analysis along a time course after adding a protein synthesis inhibitor, cycloheximide. Bay11-7082 induced a more rapid degradation rate of Myc protein (Figure 4A). Wild-type IKKα or IKKβ increased the stability of Myc protein. On the other hand, kinase-dead IKKα or IKKβ enhanced Myc protein degradation (Figure 4B and 4C).

### IKKα directly interacts with the Myc protein

Next, we used reciprocal coimmunoprecipitation followed by Western blot analysis to identify potential interactions between IKKs and Myc. The result showed that IKKα coimmunoprecitated with Myc, indicating a direct interaction between IKKα and Myc. However, the interaction between IKKβ and Myc was barely detectable (Figure 4D). The interaction between IKKα and Myc was not affected by loss of IKKα activity (Figure 4E), or by the presence of overexpressed wild-type or kinase-dead IKKβ (Figure 4F). This finding was further supported by confocal microscopy observation. The specimens from a patient with breast cancer, which stained positive for IKKα, IKKβ and Myc, were dual-stained for IKKα/Myc or IKKβ/Myc. It was observed that IKKα colocalized with Myc, whereas IKKβ and Myc was not found to be significantly colocalized (Figure 4G).

### IKKs promote anchorage-independent growth and invasiveness of MCF7 cells

**Figure 5:**
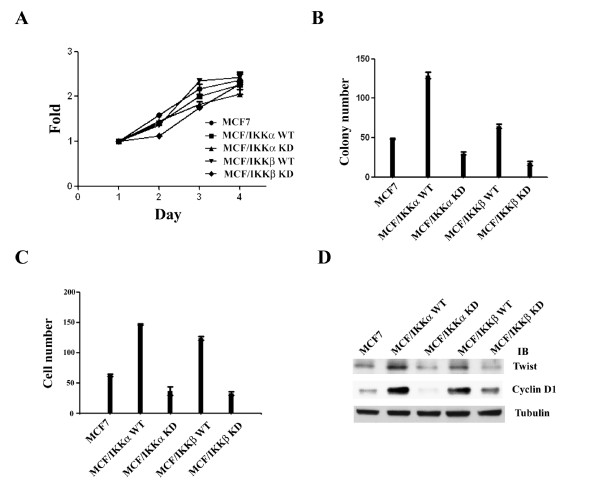
**IKKα and IKKβ increase tumorigenesis and invasiveness of MCF7 cells**. (A) MTT assay was used to analyze the growth rate of indicated cells. Each data represents mean ± SD calculated from three independent experiments, and there are four wells for each point in a single experiment. (B) Assay of colony-forming ability in soft-agar. Each experiment have three 60 mm dishes and each data represents mean ± SD calculated from two independent experiments. (C) Assay of invasive ability of indicated cells. Each experiment have three trans-wells and each data represents mean ± SD calculated from two independent experiments. (D) Western blot analysis of the expression of cyclin D1 and twist protein in the indicated cells.

We next characterized the biological functions of IKKs by analyzing IKK-transfected cells. Cells overexpressing either wild-type or kinase-dead IKKα or IKKβ showed comparable growth rates (Figure 5A). However, wild-type IKKα and IKKβ increased cell growth in soft-agar, a well-defined character of tumorigenesis, and kinase-dead IKKα and IKKβ suppressed this ability of the cells (Figure 5B). Next, we assayed the ability of IKK-overexpressing cells to pass through matrigel, a well-established method to determine the invasive activity of cancer cells. Wild-type IKKα or IKKβ overexpressing cells showed a higher invasive ability than parental MCF7 cells; by contrast, the invasive ability was decreased in kinase-dead IKKα or IKKβ-transfected cells (Figure 5C). Because cyclin D1 and Twist are two important down-stream effectors of Myc and their biological functions are associated with the invasive/tumorigenic ability of cancer cells [20,33,34], cyclin D1 and Twist protein levels were determined by Western blot analysis. Consistently, the levels of these two proteins were increased in wild-type IKKα- or IKKβ- transfected cells and decreased in kinase-dead IKKα-or IKKβ- transfected cells (Figure 5D).

### IKKs/Myc is a stress-inducible signaling pathway

**Figure 6:**
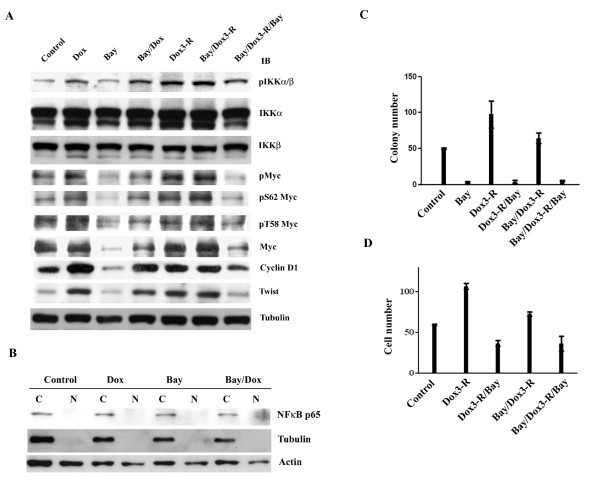
**IKKs-Myc pathway is inducible**. (A) MCF7 cells were treated with 10 μM Bay11-0782 alone, or 200 nM doxorubicin in the presence or absence of 10 μM Bay11-0782 for 24 hours (Dox, Bay, and Bay/Dox), or 3-hr short-term pulse followed by 24 hours release in the presence or absence of Bay11-0782 (Dox3-R, Bay/Dox3-R, and Bay/Dox3-R/Bay). Whole cells lysates were subjected to Western blot analysis. (B) MCF7 cells were treated 200 nM doxorubicin with or without 10 μM Bay11-0782 for 24 hours. The cytoplasmic and nuclear lysates were subjected to Western blotting. (C) MCF7 cells were treated with 200 nM doxorubicin in the presence or absence of 10 μM Bay11-0782 for 3 hours. The cells were then seeded into soft-agar in complete medium with or without 10 μM Bay11-0782 for two weeks. Each experiment have three 60 mm dishes and each data represents mean ± SD calculated from two independent experiments. (D) MCF7 cells were treated as shown, and then the ability of cells to penetrate matrigel was determined. Each experiment have three trans-wells and each data represents mean ± SD calculated from two independent experiments.

Because IKKs play an important role in tumor cells response to various stresses, it was of interest to ask whether common chemotherapeutic agents for breast cancer treatment could induce IKK and Myc activation. We used doxorubicin (200 nM; IC_50 _for MCF7 cells) alone or combined with Bay11-0782 to treat MCF7 cells. Cells were exposed to doxorubicin with or without Bay11-0782 for either 24-hour or for 3-hour followed by a 24-hour release in the presence or absence of Bay11-0782. Doxorubicin induced IKKs activation, with induction levels highest in cells released from a 3-houre doxorubicin treatment. When Bay11-0782 was re-added to the cells released from a 3-hour treatment with doxorubicin with or without Bay11-0782, decreased levels of doxorubicin-induced IKKs activation were observed. IKK activity returned to control levels after a 24-hour Bay11-0782 treatment. (Figure 6A). Consequently, the phosphorylation of Myc at Ser 62 was increased by doxorubicin, and the highest level of induction was achieved in cells released from a 3-hour doxorubicin treatment with or without Bay11-0782 cotreatment. Re-addition of Bay11-0782 blocked this induction. The phosphorylation of Myc at Thr58 remained at a relatively constant level following all treatments. The level of Myc protein was changed with a consistent pattern as the status of phosphorylated Myc Ser62. Cyclin D1 and Twist levels were also altered in a similar manner (Figure 6A). In addition, Western blot analysis showed that NF-κB was not affected by these treatments (Figure 6B).

### IKKs/Myc activated by doxorubicin promotes tumorigenesis and invasiveness of MCF7 cells

**Figure 7:**
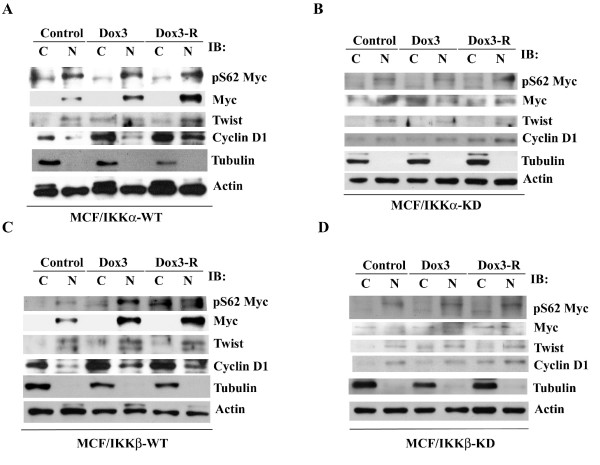
**IKK activity is necessary for doxorubicin to increase Myc, cyclin D1 and twist protein levels in MCF7 cells**. The cells (A) MCF/IKKα-WT, (B) MCF/IKKα-KD, (C) MCF/IKKβ-WT, and (D) MCF/IKKβ-KD were treated with 200 nM doxorubicin for 3hours, and then were released with complete medium for further 24 hours. The cytoplasmic and nuclear extracts were subjected to Western blot analysis.

Because cyclin D1 and Twist proteins were increased in MCF7 cells following a 3-hour doxorubicin exposure, we characterized whether the tumorigenic and invasive ability of MCF7 cells was subsequently enhanced. Indeed, the cells released from a 3-hour doxorubicin treatment increased their ability to grow in soft-agar and to pass through matrigel. Co-treatment with Bay11-0782 partially suppressed these activities, and re-addition of Bay11-0782 after released from doxorubicin markedly decreased these activities to lower than the levels of the untreated control (Figure 6C and 6D). These results were further supported by analyzing the effect of doxorubicin on IKK-transfected cells. Following the same treatments as above, the protein levels of Myc, cyclin D1, and Twist were increased in wild-type IKKα- and IKKβ-transfected cells (Figure 7A and 7C), but were not changed in kinase-dead IKKα- and IKKβ-transfected cells (Figure 7B and 7D).

## Discussion

In this study, we explored the relationship between IKKs and Myc expression in breast cancers. IHC staining of breast cancer specimens indicated that the expression of Myc was associated with IKKα and IKKβ, but was unrelated to that of NF-κB. We demonstrated that IKKα and IKKβ did not enhance Myc transcription but instead increased Myc protein stability. Importantly, we also showed that the commonly used anticancer drug, doxorubicin, activated IKKs, and thereby increased Myc protein levels. Myc plays an important role in tumor progression and is associated with metastasis and a poor outcome of breast cancers [7,17,19]. However, there is still no reliable drug which can effectively target Myc. Our study indicates that one possible way to block Myc is by inhibition of IKKs.

It is well known that IKKs trigger IκBα degradation and subsequent activation of NF-κB [10]. However, our results showed that IKKα and IKKβ regulated Myc expression levels without altering NF-κB activation. NF-κB activation is tightly auto-regulated by inducing the expression of its natural inhibitor, IκBα. For example, in response to TNFα stimulation, IκBα is degraded during the first 15 minutes and quickly restored in one hour. Therefore, we hypothesized that transfection of wild-type IKKα and IKKβ may have transiently activate NF-κB, but that the activity of NF-κB subsequently returned to basal levels due to the balance between NF-κB and IκBα.

In this study, we showed that suppression of IKK activity by transfection of kinase-dead IKKα or IKKβ decreased Myc protein levels, whereas slightly increased the Myc mRNA levels. Our results demonstrated that the change in Myc protein levels was not related to the transcription of Myc, consistent with a previous study performed in neuroblastoma [35]. Our study further demonstrated that the turn-over rate of the Myc mRNA was prolonged in kinase-dead IKKα- and IKKβ-transfected cells. Interestingly, Bay11-0782 did not show this activity, suggesting that it may have other effect on mRNA stability. While the underlying mechanism remains unknown, our result may help explain the conflicting results in analyzing the relative levels of mRNA and protein of certain genes.

The stability of Myc is controlled by its phosphorylation at Ser62 and Thr58 [16]. In this study, we showed that IKKα and IKKβ increased Myc protein stability by regulating its phosphorylation status at Ser62. We further demonstrated that IKKα but not IKKβ directly interacted with Myc. While the pathway connecting IKKβ to Myc remains to be identified, our study demonstrated a reciprocal upregulation between IKKα and IKKβ, indicating that IKKβ may indirectly increase Myc through IKKα. However, it is of particular interest to determine whether IKKβ may also affect Myc through regulation of related molecules, such as PP2A or cip2A.

A comparable growth rate was observed among parental MCF7 cells and IKKs-transfected cells. Because Bay11-0872 indeed decreased MCF cell growth [data not shown], it is likely that un-identified pathways which can compensate for Myc activity are developed during the selection of kinase-dead IKKα- and IKKβ-transfected cells. In addition, our result indicated that the invasive ability induced by Myc could be separated from the growth potential of cancer cells and that Myc activity was indispensable for the enhanced invasiveness. Our results are consistent with the data from a previous study using another breast cancer cell line MDA-MB 231 [17].

Based on gene array analysis, Myc has been shown to regulate a set of gene signatures associated with metastasis and poor-outcome of breast cancers [17]. Abnormal expression of Myc promotes the epithelial to mesenchymal transition and metastasis [14,18,36]. In this study, we showed that Myc increased the tumorigenic and invasive ability of MCF7 cells. We also identified that the levels of cyclin D1 and Twist were consistently altered along with the Myc protein level. It is reasonable to conclude that Myc enhances tumorigenesis, at least in part, through the upregulation of cyclin D1 and Twist. However, overexpression of Myc in a non-invasive, transformed breast cell line [MCF10A] does not promote its invasive ability, suggesting that Myc is a necessary but not sufficient factor for cancer cell invasiveness [22]. It is hypothesized that Myc should cooperate with other factors to enhance the invasive activity of the cells. In this study, Myc was increased downstream of IKKα and IKKβ activation. Both IKKα and IKKβ also regulate the expression of multiple genes that are involved in cancer cell progression and metastasis. Therefore, it is possible that a Myc-centered network cooperates and/or merges with an IKK-centered network to enhance the tumorigenic and invasive activity of cancer cells. These complicated interactions should be further characterized through systemic genetic studies.

In this study, we provided evidence that doxorubicin increased Myc protein levels probably through activating IKKα and IKKβ. The cells that were released from doxorubicin increased their invasive and tumorigenic activities, and suppression of IKK activation blocked these phenotypes. Myc regulates many downstream gene expressions which share a similarity between embryonic stem cells and cancer cells [37,38]. It is important to determine whether doxorubicin stimulates re-growth and progression of cancer cells *in vivo*. On the other hand, IKKs are major mediators linking inflammation and cancer progression [10,39]. We have not tested other stimulus, such as inflammation-related cytokines, that may have similar effects on the IKK/Myc pathway; however, it is possible that the death of cancer cells caused by therapeutic treatment may trigger an inflammation response, which activates IKKs and Myc in the remaining cancer cells, and subsequently stimulates cancer progression. In addition, IKKs and Myc are widely expressed in various cancers. For example, IKK and Myc have been separately reported to be necessary for hepatocellular carcinoma cell growth and invasiveness [2,8]; it is therefore likely that our finding may be applicable to other cancers. Taken together, our results suggested that IKKs/Myc might be important therapeutic targets for breast cancer and provided a rationale for the use of IKK inhibitors following chemotherapy to suppress the treatment-enhanced tumor progression.

## Competing interests

The authors declare that they have no competing interests.

## Authors' contributions

PY designed and carried out the cellular and molecular studies, and wrote the manuscript, YS carried out the clinical studies, DL carried out the gene expression studies, and AL organized and supervised the whole study. All authors read and approved the final manuscript.
